# Non-invasive estimation of QLV from the standard 12-lead ECG in patients with left bundle branch block

**DOI:** 10.3389/fphys.2022.939240

**Published:** 2022-09-21

**Authors:** Jacob Melgaard, Peter M. van Dam, Anders Sommer, Patricia Fruelund, Jens Cosedis Nielsen, Sam Riahi, Claus Graff

**Affiliations:** ^1^ CardioTech Research Group, Department of Health Science and Technology, Faculty of Medicine, Aalborg University, Aalborg, Denmark; ^2^ Department of Cardiology, University Medical Center Utrecht, Utrecht, Netherlands; ^3^ Peacs BV, Nieuwerbrug Aan Den Rijn, Netherlands; ^4^ Department of Cardiology, Aalborg University Hospital, Aalborg, Denmark; ^5^ Department of Cardiology, Aarhus University Hospital, Aarhus, Denmark

**Keywords:** cardiac modeling, electrophysiology, ventricular activation, left bundle branch block, cardiac resynchronization therapy

## Abstract

**Background:** Cardiac resynchronization therapy (CRT) is a treatment for patients with heart failure and electrical dyssynchrony, i.e., left bundle branch block (LBBB) ECG pattern. CRT resynchronizes ventricular contraction with a right ventricle (RV) and a left ventricle (LV) pacemaker lead. Positioning the LV lead in the latest electrically activated region (measured from Q wave onset in the ECG to LV sensing by the left pacemaker electrode [QLV]) is associated with favorable outcome. However, optimal LV lead placement is limited by coronary venous anatomy and the inability to measure QLV non-invasively before implantation. We propose a novel non-invasive method for estimating QLV in sinus-rhythm from the standard 12-lead ECG.

**Methods:** We obtained 12-lead ECG, LV electrograms and LV lead position in a standard LV 17-segment model from procedural recordings from 135 standard CRT recipients. QLV duration was measured post-operatively. Using a generic heart geometry and corresponding forward model for ECG computation, the electrical activation pattern of the heart was fitted to best match the 12-lead ECG in an iterative optimization procedure. This procedure initialized six activation sites associated with the His-Purkinje system. The initial timing of each site was based on the directions of the vectorcardiogram (VCG). Timing and position of the sites were then changed iteratively to improve the match between simulated and measured ECG. Noninvasive estimation of QLV was done by calculating the time difference between Q-onset on the computed ECG and the activation time corresponding to centroidal epicardial activation time of the segment where the LV electrode is positioned. The estimated QLV was compared to the measured QLV. Further, the distance between the actual LV position and the estimated LV position was computed from the generic ventricular model.

**Results:** On average there was no difference between QLV measured from procedural recordings and non-invasive estimation of QLV ( $\Delta_{QLV}=-3.0\pm 22.5\;ms,\;p=0.12$ ). Median distance between actual LV pacing site and the estimated pacing site was 18.6 mm (IQR 17.3 mm).

**Conclusion:** Using the standard 12-lead ECG and a generic heart model it is possible to accurately estimate QLV. This method may potentially be used to support patient selection, optimize implant procedures, and to simulate optimal stimulation parameters prior to pacemaker implantation.

## Introduction

Cardiac resynchronization therapy (CRT) is a treatment option intended for patients with heart failure and reduced ejection fraction (HFrEF) and wide QRS on the electrocardiogram (ECG) (Glikson et al., 2021). The therapy was developed in the 1990s (Leclercq et al., 1998) and gained widespread use in the early 2000s. It is known that response varies greatly from patient to patient, with some improving much in both symptoms and survival, while others see no or even a negative response (Ypenburg et al., 2009). This has entailed great interest in finding markers for positive response. Initially, a stricter definition of the ECG criteria for left bundle branch block (LBBB) was proposed (Strauss et al., 2011), but still, about one third of these patients do not experience improvement in symptoms. Lack of response is most likely multifactorial, including patient selection, presence of large areas of myocardial scarring, implantation site of the left ventricular (LV) pacing lead, and timing of the stimulus impulse. Further, CRT has been shown to be beneficial in some subgroups of patients with HF and non-LBBB ECG, e.g. Right Bundle Branch Block (RBBB) or non-specific Intra-Ventricular Conduction Delay (IVCD), stressing that other factors than solely QRS duration or LBBB morphology determine outcome (Salden et al., 2020). Therefore, more knowledge about the activation sequence of the heart prior to CRT implantation may impact selection of the best CRT candidates and be valuable in planning CRT implantation.

Inverse ECG computer modeling is a means for relating ECG signals measured on the body surface to the cardiac electrical activation through equations governed by physical laws. Computing the ECG from a cardiac activation pattern is termed the forward problem, while going from ECG to myocardial activation is termed the inverse problem, or often simply electrocardiographic imaging (ECGi). The inverse problem in electrocardiography cannot be solved uniquely, and generally recordings from much more than the 10 electrodes used for measuring the 12-lead ECG are used when solving the inverse problem. Such recordings are termed body surface potential maps (BSPM). A computationally efficient way of solving the inverse problem is to define a fixed set of activation sites and minimize the error between simulated and measured ECGs or BSPMs (van Dam, Oostendorp, Linnenbank, et al., 2009). Given fixed activation sites, forward models have been able to accurately simulate LBBB ECGs (Galeotti et al., 2013; van Dam et al., 2014). Similarly, a fastest route algorithm optimizing the activation pattern with regard to the error between the simulated and measured 12-lead ECG, by moving and delaying a number of activation sites, was able to simulate the ECG accurately (Roudijk et al., 2021; Boonstra et al., 2022). Ideally, an individual geometric model should be made for each patient, to improve simulation accuracy. However, this requires CT or MRI scans of the patient, and time-consuming manual work to segment the heart, lungs and thorax. Hence, this is often not possible in standard clinical practice.

In this study we use an inverse ECG algorithm together with a generic geometric model and the standard 12-lead surface ECG to estimate the activation sequence of the heart for LBBB patients. Further, we estimate QLV from a given anatomical location and compare with procedurally measured QLV in patients with sinus rhythm and LBBB undergoing CRT implantation.

## Methods

### Study population

The study population consisted of 182 patients included in the Empiric Versus Imaging Guided Left Ventricular Lead Placement in Cardiac Resynchronization Therapy (ImagingCRT) study (Clinical Trials record NCT01323686); a double-blinded, randomized controlled trial. The design and results of the study have previously been published (Sommer et al., 2013, 2016). Briefly, the study investigated if imaging guided optimal left ventricular (LV) lead placement targeting the latest mechanically activated non-scared segment improved the response rate to cardiac resynchronization therapy (CRT) compared with standard LV lead placement. A 12-lead surface ECG and bipolar electrograms were recorded during the implantation procedure (CardioLab, GE Medical, Milwaukee, MN). At the time of collection, 31 records could not be retrieved from CardioLab, 15 records had no sinus rhythm ECG (patients were pacemaker dependent), and one showed a normal QRS duration of 84 ms and was therefore excluded. The remaining 135 patients were included in this study.

The included 135 patients all had simultaneous 12-lead surface ECG and bipolar right ventricular (RV) and LV pacing lead electrograms measured at the final implant site. Data was exported as two paired files per patient, one with the 12-lead ECG, and one where lead V1 was exchanged for the bipolar LV sense signal (ECG/LV). QRS duration was measured manually by one reviewer (C.G.) with digital calipers on the ECGs with magnification 200%, and the QRS onset and offset points were transferred to the ECG/LV files. QLV was then measured on the ECG/LV files by two reviewers (C.G. and J.M.), also using a digital caliper at a magnification of 500%. Any discrepancies were resolved by consensus. AHA17 segment location (Cerqueira et al., 2002) for the LV electrode was evaluated by post-implant cardiac computed tomography (A.S.) and was available for all 135 patients from study records.

### QLV measurement

**FIGURE 1:**
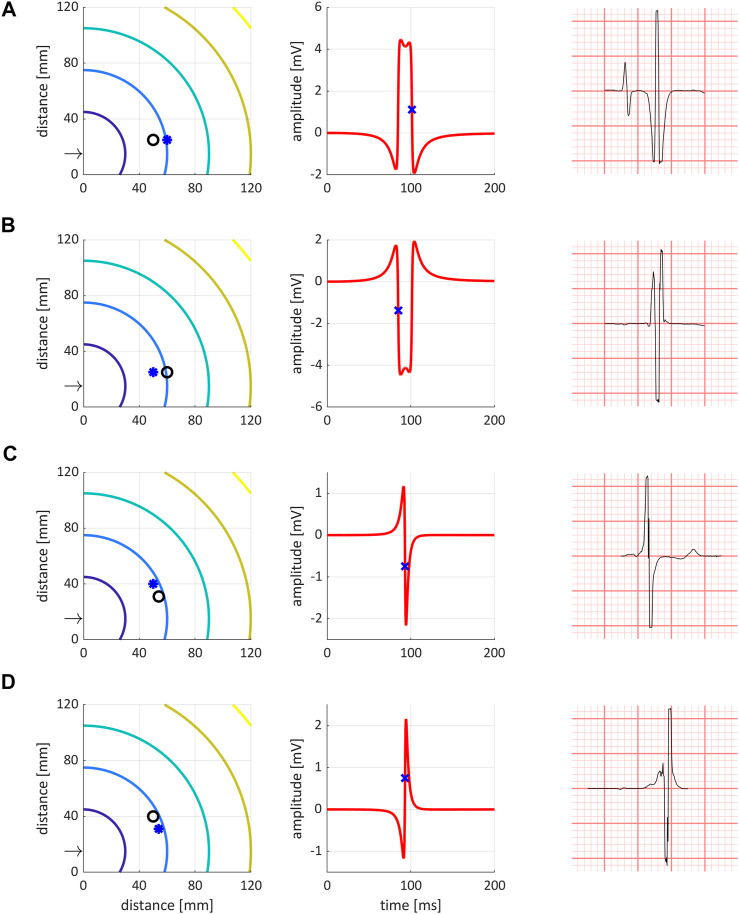
The four bipolar electrogram phenotypes identified and simulated. Left column are simulated isochrones, with the ring denoting the negative electrode, and the star denoting the positive catheter tip electrode. The arrow points to the stimulation site at (*x* = 0 mm, *y* = 15 mm). Middle column shows simulated bipolar electrograms. Right column shows representative examples from the data. **(A–D)** (horizontal) show the four different configurations. Please refer to the text for further details.

The morphology of bipolar electrograms depend on both the direction of the activation wave front and the tissue-electrode distances (Bakker, 2019). We identified four different QLV morphology phenotypes in our data. Bipolar electrograms are simulated using a moving circular dipole layer in an infinite medium approximated by triangulated rings. Simulations were done in an infinite 3D slab of homogenous isotropic tissue with thickness of 4 mm. We visualize the wavefront only for a 120 mm by 120 mm region of this slab. The simulated sensing catheter has electrode spacing of 10 mm. The stimulus origin was at *x* = 0 mm and *y* = 15 mm (referenced to the visualized region), with the catheter electrodes placed approximately 40 mm away from this origin. We first computed the activation map analytically with a conduction velocity of 0.6 m/s. Then, with a time sampling of 0.1 ms, we computed the total solid angle (van Oosterom and Strackee, 1983) per time step of the triangulated circular ring (400 triangles); this being a scaled version of the local infinite medium potential at one measuring electrode (the unipolar electrogram). The bipolar electrogram is then the subtraction of the two simulated electrograms from the two differently located electrode positions. As the electrodes are relatively close together, the approximation of the volume conductor as being an infinite medium is approximately valid. A limitation of these simulations is that the local heart curvature and anisotropy is not taken into account, because this data was not available Also, bath loading effects were not including is this simulation. For different electrode orientations the electrogram could be simulated as shown in Figure 1. One bipolar electrogram simulation takes approximately 4 s on a standard office pc (Intel core i7 3 GHz running Windows). Panel (A) shows propagation along the catheter direction, with the tip closest to activation origin. In this case, activation at the catheter tip causes the first negative deflection in the bipolar ECG. Hence, to reflect local activation time (LAT) at the catheter tip, we measure the first negative slope of the signal. Panel (B) also shows propagation along the catheter, but in the opposite direction. In this case, the second leg (but still the steepest negative slope) represents LAT at the tip. Panel (C) shows electrodes nearly perpendicular to the activation wavefront, giving rise to a biphasic signal. In this case, the steepest negative slope is measured as representative for LAT at the electrode tip. Finally, panel (D) shows a situation similar to (C), but with the electrodes interchanged with respect to the activation wavefront. In this case, the steepest positive deflection is representative of LAT at the tip. The four phenotypes are denoted configuration one to four, for (A)-(D), respectively.

To reflect local activation time (LAT) at the catheter tip, all EGMs were first classified as one of the four phenotypes, and based on this configuration the QLV was determined accordingly.

### The mapper

**FIGURE 2:**
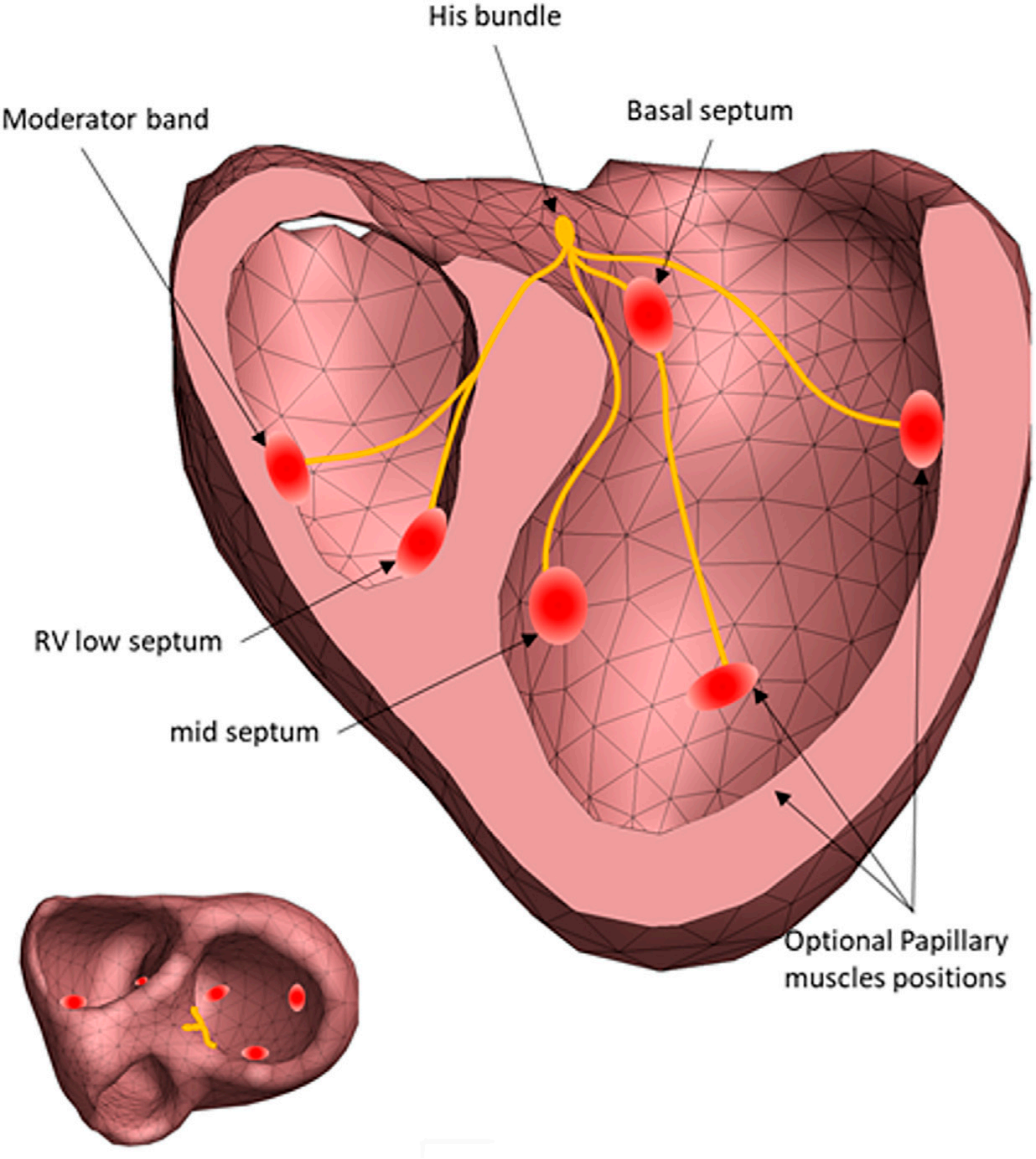
The regional sites of early activation associated with the Purkinje activation. Each red circle identifies a position of the initial estimation of the cardiac activation. The exact position of the His-Purkinje system is unknow, only the effect of the His-Purkinje system on cardiac activation is simulated (left panel).^15^.

The Mapper is a modeling approach that aims to estimate the cardiac activation initiated from the His-Purkinje system by optimizing a forward solution to best match the ECG. The mapper has previously been validated using both endocardial and epicardial recordings (Roudijk et al., 2021; Boonstra et al., 2022), and has also been shown to accurately show PVC foci (Potyagaylo et al., 2019) and general morphological changes occurring with LBBB (Galeotti et al., 2013). The steps involved in The Mapper algorithm are shown in Figure 2. Briefly, a generic geometry and a patient-specific ECG are used as input. Using this data, the major QRS axis is computed. Depending on the QRS axis and QRS duration, an initial activation time is set for each of the “His-Purkinje” nodes, or the nodes are “disabled”. In the final optimization step, timing and position of the “His-Purkinje” nodes are changed to best fit the patient specific ECG. The human His-Purkinje system distributes the electrical activation to a large part of the endocardial surface of the myocardium. In this study the initial activation from a branch of the His-Purkinje system is approximated by an endocardial surface being activated almost simultaneously, attributed to the density of the local available Purkinje-myocardial junctions located on the endocardial surface. Thus, the Purkinje initiated ventricular activation is modelled by a combination of multiple breakthroughs in different parts of the left and right ventricular myocardium.

#### Activation sequence construction

The fastest route algorithm is used to compute the activation propagation from the initial sites of activation (van Oosterom and van Dam, 2005; van Dam, Oostendorp, and van Oosterom, 2009b). The fastest route algorithm computes the (virtual) distances in the geometric heart model between a node and all other nodes on a closed triangulated myocardial surface. The propagation velocity within the myocardium is anisotropic, i.e., velocities perpendicular to the myocardial fiber direction is about 2 times slower than along the fiber direction. To take this anisotropic propagation velocity into account the (virtual) distance for transmural connections is made 2.5 times longer, as the transmural connections are by definition perpendicular to the local fiber direction (van Dam, Oostendorp, and van Oosterom, 2009b; van Dam, Oostendorp, Linnenbank, et al., 2009). To mimic the surface activation from the Purkinje system, the local velocity around a node on the ventricular surface is set to 1.7 m/s with a radius of 15 mm. The Mapper is described in detail by Roudijk et al. (Roudijk et al., 2021), especially in the supplementary material, and by Boonstra et al. (Boonstra et al., 2022).

**FIGURE 3:**
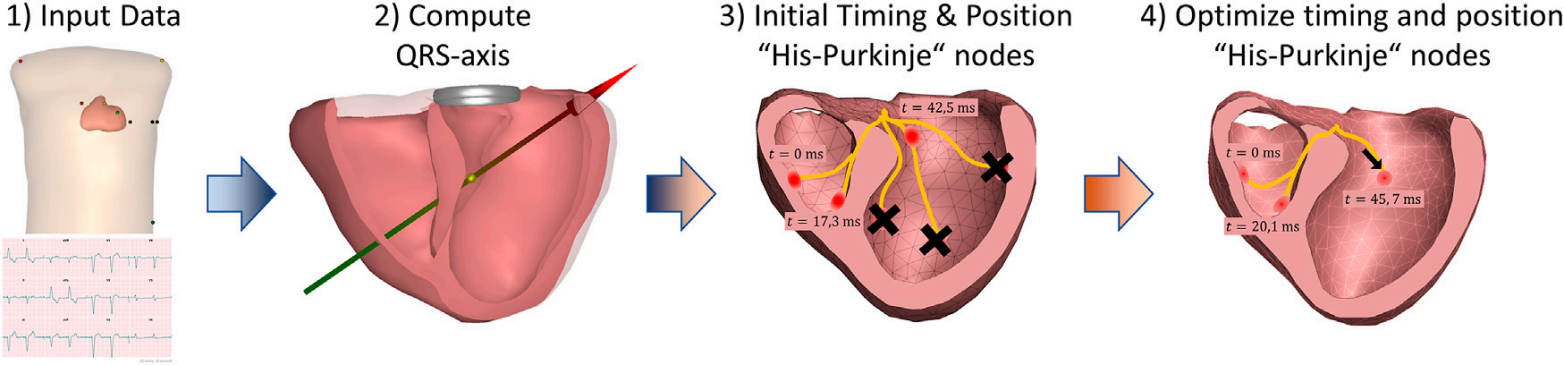
The steps involved in The Mapper algorithm. 1) First, a geometry (generic or patient specific) and a patient specific 12-lead ECG is used as input. 2) Initial analysis of the QRS axis and QRS duration determines gross activation pattern, and based on this 3) pre-defined times are set for the six His-Purkinje activation sites (or sites are “disabled”). Finally, 4) both the position and the activation times of the His-Purkinje activation sites are changed to best fit the ECG.

The geometric model used in this study to estimate the cardiac activation is from a 58-year-old male which we find to have typical body build and heart orientation. The source method used to simulate the equivalence of the cardiac activity is the equivalent dipole layer (EDL) (van Dam, Oostendorp, & van Oosterom, 2009a; van Dam, Oostendorp, Linnenbank, et al., 2009; van Oosterom, 2011; van Dam et al., 2013; Janssen et al., 2018). This model is used to compute the ECG given the activation time at each of the nodes of the ventricular mesh (Figure 3). Several methods exist to account for the volume conductor effect and compute the ECG from local activation times, for instance the Lead Field approach (Potse, 2018), or the commonly used Boundary Element Method. In this research the Boundary Element Method was used. The volume conductor model uses the geometries of the thorax, ventricles, and ventricular blood cavities. The conductivity of the blood was set to be 3 times the value of the rest of the thorax geometry (Roudijk et al., 2021).

#### Phenomenological estimation of His-Purkinje system activation

To estimate the His-Purkinje activation sequence from a patient’s ECG, an initial activation sequence is generated to simulate ECG signals. This initial sequence uses six different anatomical locations of potential Purkinje activations sites (Figure 2), based qualitatively of the activation sequence described by Durrer et al. They described that initial activation was typically found on the anterior left septum, with later local breakthroughs in the left and right apical regions as well as the right free wall (Durrer et al., 1970). Hence, for the LV septum, two sites are located basal and mid septum. Two more locations were selected on the left free wall, associated with papillary muscle locations and thus with potential sites of early His-Purkinje activation. The two positions on the endocardial RV wall represent the entry of the moderator band (Ho et al., 2003) associated with the papillary muscles, and a site at the right apical septum. This approach was previously validated (Roudijk et al., 2021; Boonstra et al., 2022).

The initial timing of each of these six locations depend on the morphology of an ECG waveform and they are used as an initial guess for subsequent iterative optimization procedures. For normal ECG morphology, the initiation times of the left septal wall was set to 0 ms, i.e. equal to the QRS onset, while the RV and LV initiation times are set to 15 ms. For ECGs with an LBBB pattern (QRS duration >120 ms) the initial timing of the left regions, is delayed to 40 ms for the septal regions and 45 ms for the free wall regions. Similarly, for RBBB ECG waveforms (QRS duration >120 ms), the initial activation of the RV septal region is set to 45 ms, and the RV free wall to 65 ms.

In the subsequent optimization procedure, the timing and position of these six early sites of activation can be changed to obtain the best match between the simulated ECG and measured ECG. The total activation duration for each constructed sequence is matched to the QRS duration by adapting the overall used propagation velocity. The used propagation velocity is maintained within the physiological range of the myocardial velocity, i.e., between 0.6 and 0.85 m/s (Roberts et al., 1979; Spach and Kootsey, 1983; Kléber and Rudy, 2004; Cantwell et al., 2015; Good et al., 2020).

### Analysis of activation times and LV pacing site

For each patient, the mapper estimates the activation sequence of the heart based on the 12-lead ECG. The mesh-model of the heart is divided into the AHA 17 defined segments on a node level, and the geometric mean of the segment containing the LV pace electrode is computed. Since the pacing site within an AHA 17 segment is not known, we set the LV pacing site to be in the center of the myocardial segment assessed by cardiac computed tomography. The estimated activation time at this location is found by linear interpolation within the triangle encompassing the geometric mean of the segment. The time difference between the measured and estimated activation time, denoted $\Delta_{QLV}$ , is given in milliseconds.

From the estimated activation of the whole heart, we also determined the distance between the LV electrode position (defined and computed as above) and the closest point which is estimated to activate at the measured QLV time. This is done by first searching for the nearest triangulated surface element containing an activation time equal to the measured QLV. For this triangular element, the precise location activating at this time is found by linear interpolation within the triangle. All distances between points are given in millimeters. Finally, we compared the segment of the estimated QLV position to the segment containing the LV lead. We report the fraction that is placed within the same segment.

### Statistical analysis

Continuous variables are reported as mean ± SD unless severely skewed. Categorical values are reported as absolute numbers and percentages. For analysing associations, unpaired t -test was used for continuous independent variables. p<0.05 was considered statistically significant. Matlab 2021b was used to perform the statistical analyses.

## Results

Baseline patient characteristics are shown in Table 1. The Mapper successfully estimated activation sequences for all patients. The mean difference between estimated and measured QRS duration was −0.7±3.3 ms (mean ± SD). For 125 cases out of the 135, the QRS difference was within −2 ms to +4 ms; the remaining 10 cases were outliers with a difference between −2 and −20 ms. Median correlation between simulated and recorded ECGs was 0.73. The Mapper runs in near real-time, with an execution time of approximately 1 s (and always less than 2 s) on a standard office PC (2018 Intel core i7, 3 GHz) running Windows.

**TABLE 1:** Baseline characteristics.

	All
Subjects, n	135
Female sex, n (%)	32 (24)
Age	70 ± 9 years
Height	174 ± 9 cm
Weight	80 ± 16 kg
BMI	26.3 ± 4.5 kg m^−2^
Ischemic Heart Disease, n (%)	63 (47)
Pacemaker, n (%)	6 (4)
QRS Duration	163 ± 21 ms
QLV	135 ± 28.6 ms
LVEF	25 ± 5.7%
LV EDF	259 ± 83.3 ml
LV ESV	196 ± 71.2 ml

**FIGURE 4:**
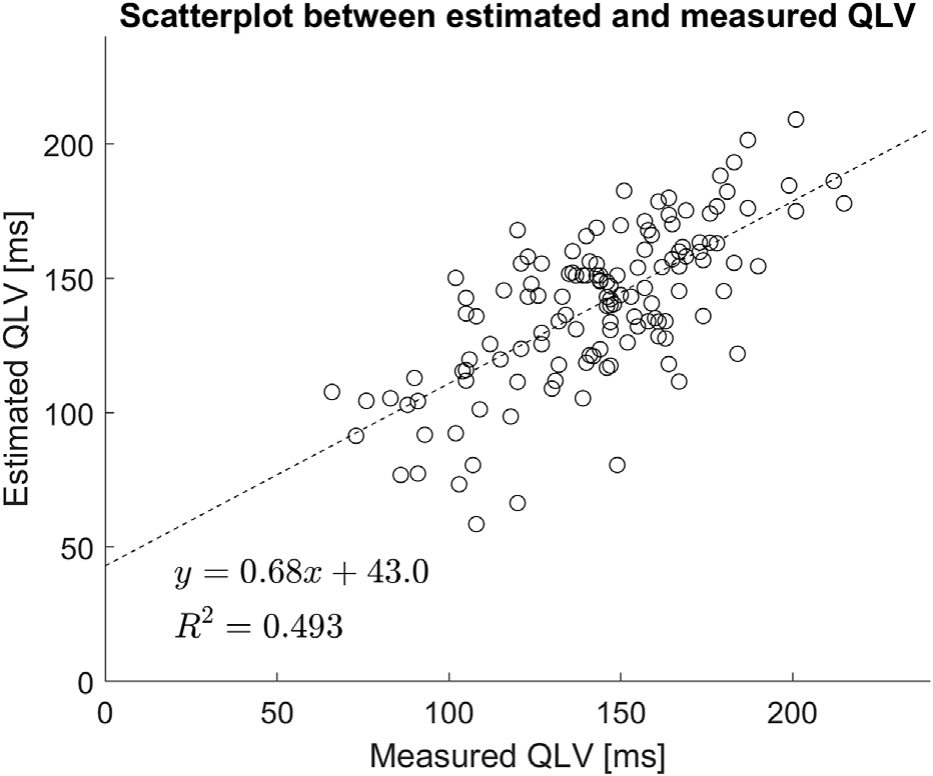
Scatter plot of estimated vs. measured QLV. Included is also a linear regression analysis.

**FIGURE 5:**
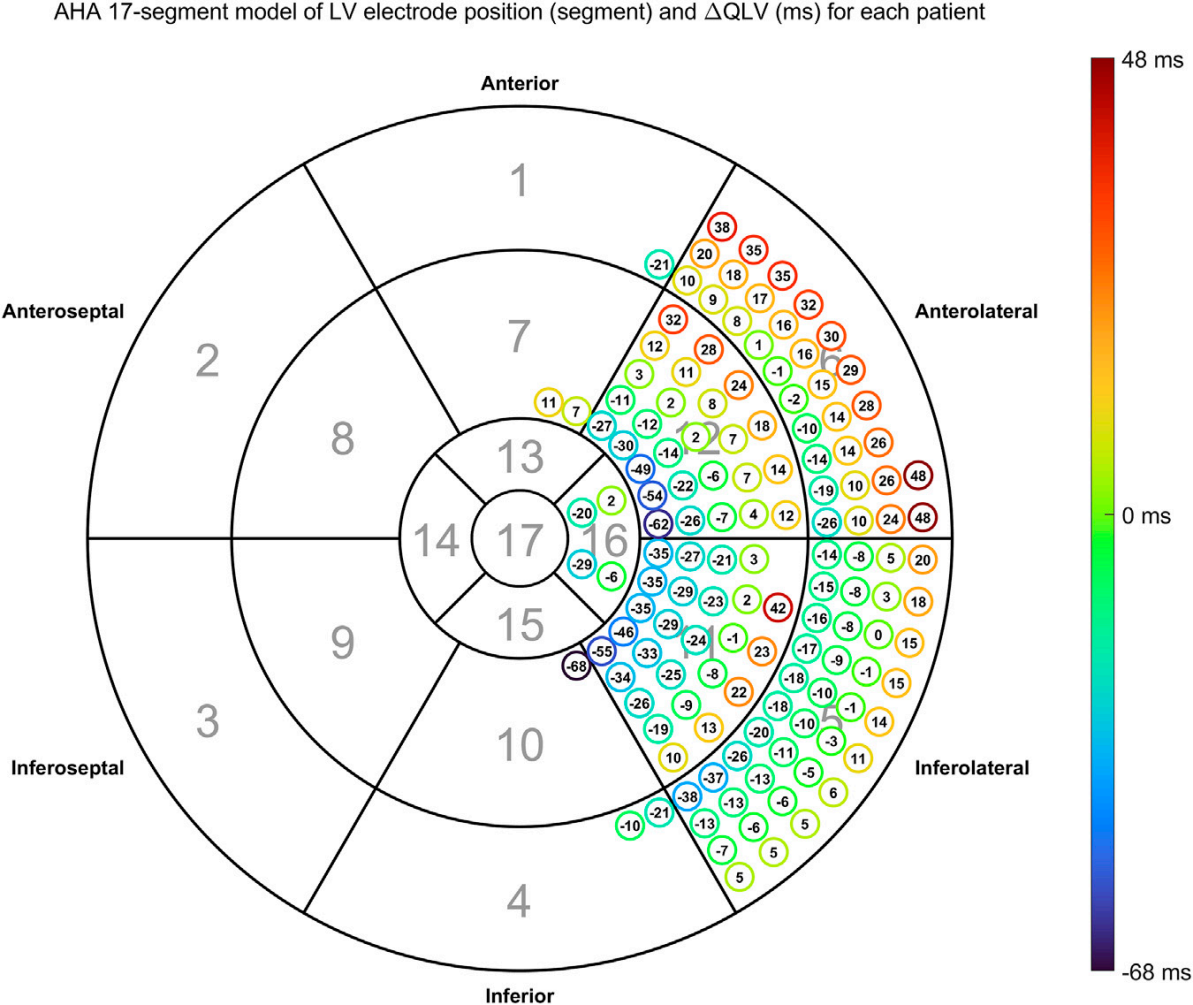
Time difference between estimated and measured QLV ( $\Delta_{QLV}$ ) according to anatomical LV lead position. The encircled numbers show $\Delta_{QLV}$ for each patient. The color of the circle is a visual representation of the $\Delta_{QLV}$ value.

On average there was a small and not statistically significant difference between measured and estimated QLV ( $\Delta_{QLV}=-3.0\pm 22.5\;ms$ ; mean ± SD, p = 0.119). Regression between estimated and measured QLV is highly significant and shows $R^{2}=0.49$ . A scatterplot together with the regression line is shown in Figure 4. All estimated QLV values are shown in the AHA 17-segment illustration in Figure 5.

Overall distribution of LV pacing site given as AHA 17 segments is shown in Table 2, while overall distribution of EGM configurations is shown in Table 3. The overall median distance between measured and estimated LV pacing site was 18.6 mm (IQR = 17.3 mm). In Tables 2, 3 we also present the fraction of correctly estimated QLV segments.

**TABLE 2:** Overall distribution of LV pacing site in terms of AHA17 segments and associated $\Delta_{QLV}$ .

LV pacing site [AHA17 segment]	n	$\Delta_{QLV}$	p -value	Mean distance [mm]	Fraction correct
		μ (±SD) [ms]			
1	1	-21		17	1/1 (100%)
4	2	-15		25	1/2 (50%)
5	40	-6 ± 14	0.012	15	32/40 (80%)
6	32	16 ± 18	0.000	22	24/32 (75%)
7	2	+9		12	2/2 (100%)
10	1	-68		58	0/1 (0%)
11	26	-15 ± 23	0.003	28	8/26 (31%)
12	27	-5 ± 24	0.294	23	15/27 (56%)
16	4	-13		18	3/4 (75%)

n : number of patients with LV pacing site at the given segment. $\Delta_{QLV}$ μ and SD: mean and standard deviation of $\Delta_{QLV}$ for all patients with LV pacing site in the given segment. In groups with four patients or less, no standard deviation was computed, and hence no *p*-value could be computed. p -value: test for the hypothesis μ=0. Mean distance: distance between LV pacing site and the nearest site estimated to activate at the measured QLV time. Fraction correct: The fraction of estimated QLV locations in the same segment as LV lead. Please refer to Figure 3 or five for anatomical location of AHA segment number.

**TABLE 3:** Overall distribution of EGM configurations and associated $\Delta_{QLV}$ .

Configuration	n	$\Delta_{QLV}$	p	Mean distance [mm]	Fraction correct
		μ±SD [ms]			
1	24	-8 ± 24	0.101	23	15/24 (62%)
2	19	+8 ± 22	0.137	19	13/19 (68%)
3	45	-4 ± 25	0.294	23	28/45 (62%)
4	47	-4 ± 19	0.151	19	30/47 (64%)

n : number of patients with ECG configuration as defined in Figure 1. $\Delta_{QLV}$ μ and SD: mean and standard deviation of $\Delta_{QLV}$ for all patients with LV pacing site in the given segment. In groups with four patients or less, no standard deviation was computed, and hence no *p*-value could be computed. p -value: test for the hypothesis μ=0. Mean distance: distance between LV pacing site and the nearest site estimated to activate at the measured QLV time. Fraction correct: The fraction of estimated QLV locations in the same segment as LV lead.

**FIGURE 6:**
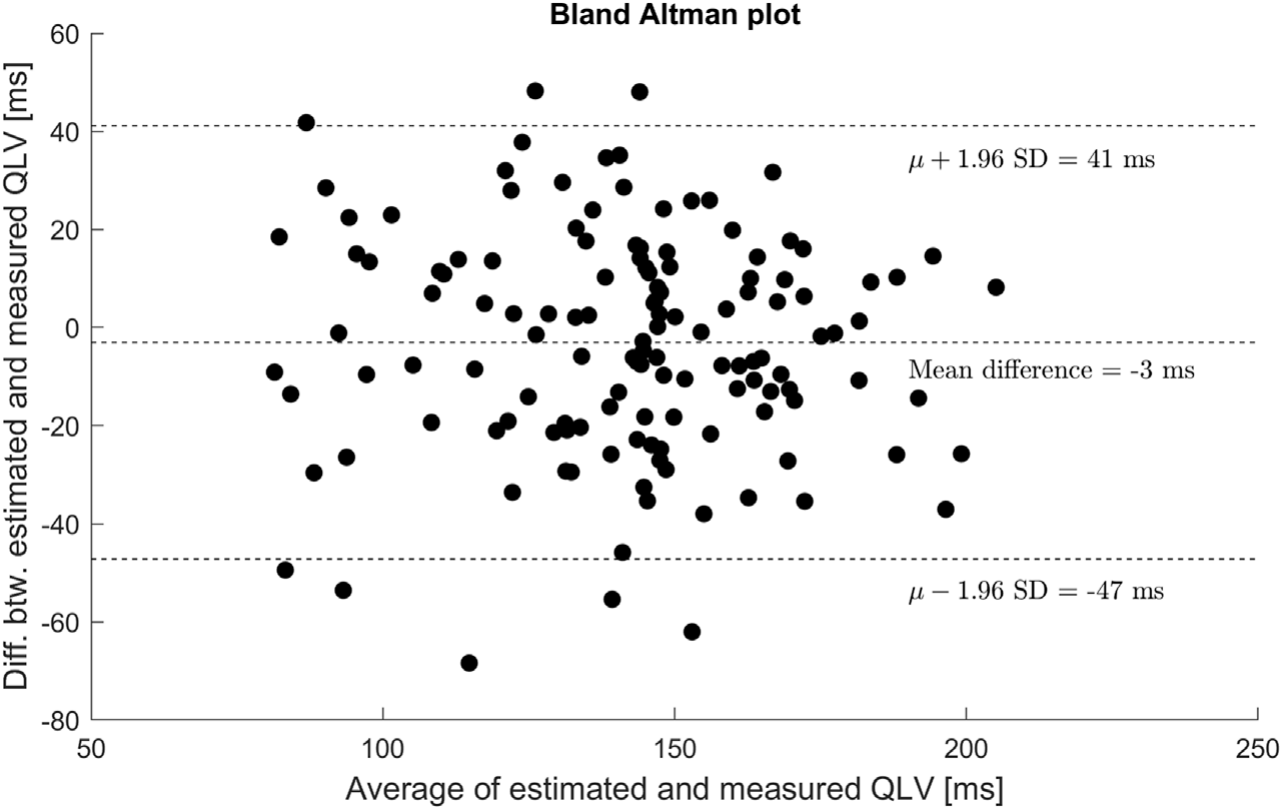
Bland-Altman plot showing agreement between estimated and measured QLV. No general trends were identified from the plot.

Similarly, there was no systematic difference or proportional bias between measured QLV and $\Delta_{QLV}$ (Figure 6).

**FIGURE 7:**
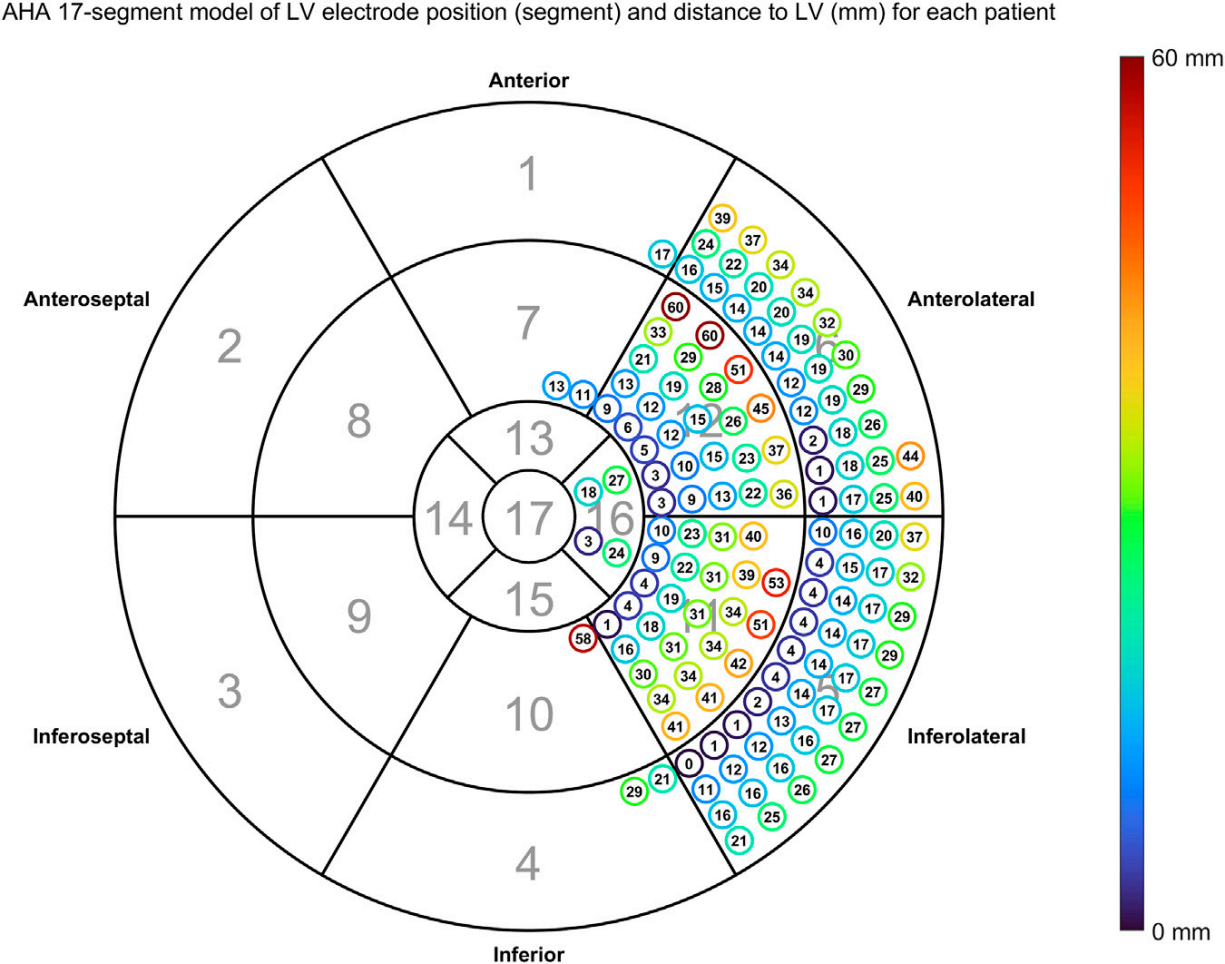
Distance between anatomical LV pacing site and nearest node activating at the measured $\Delta_{QLV}$ . Each encircled number shows the computed distance between estimated position and position determined by physician. The color of a circle is a visual representation of these distances.

Figure 7 shows the distances between LV pacing site and the nearest point activating at the measured QLV time also using the AHA 17-segment model.

**FIGURE 8:**
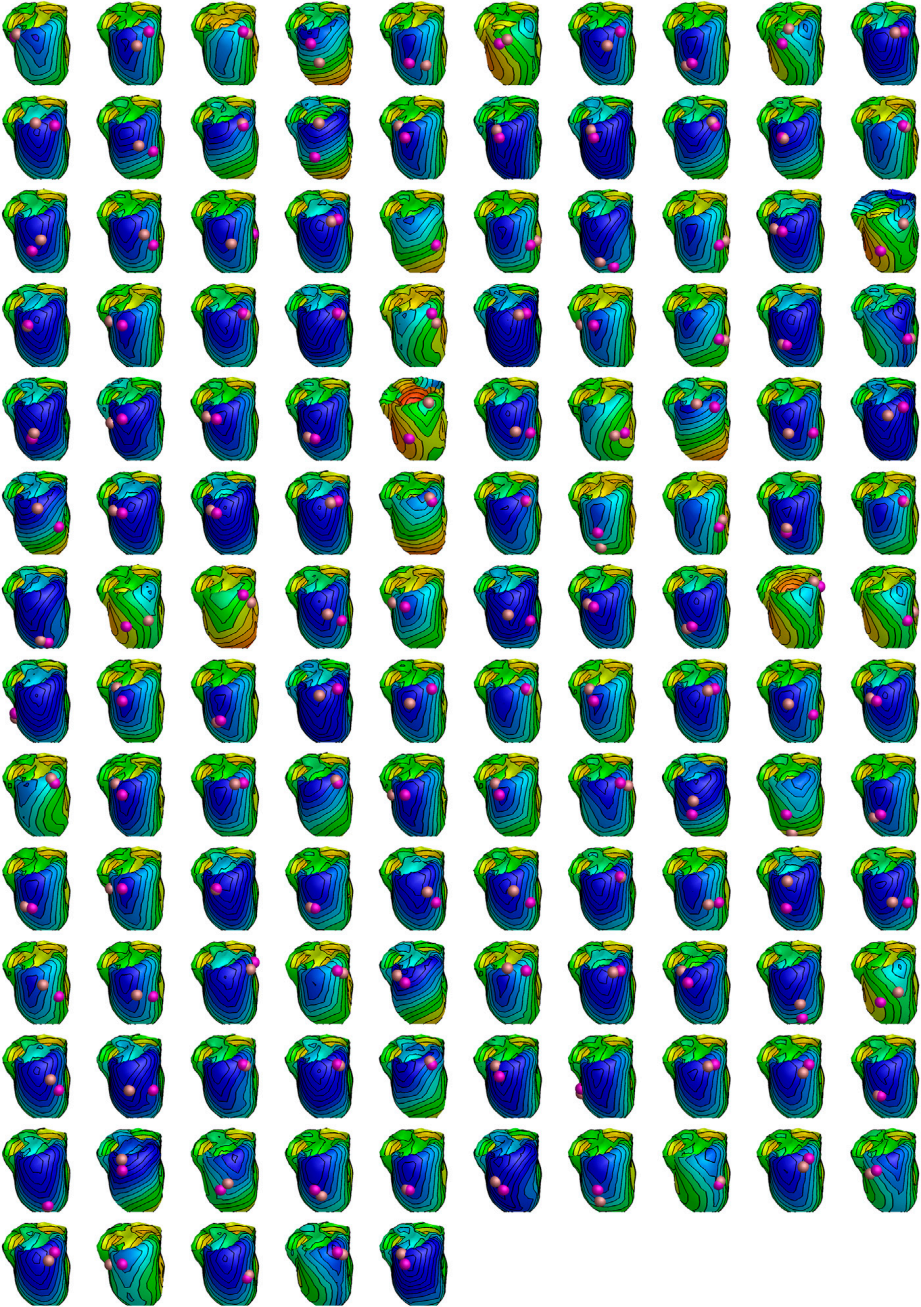
Estimated activation patterns for all 135 patients in the study. All hearts are oriented similarly independent of LV pacing site. Early to late activation is coded with red (earliest) over yellow and green to blue (latest) colors. The coloring is normalized to the patient specific QRS duration. The purple sphere shows the LV pacing site determined by physician by cardiac computed tomography (always in the center of a segment), while the bronze-colored sphere shows the nearest site activating at the measured QLV time. Both spheres are drawn with a diameter of 8 mm.

Figure 8 illustrates, for all 135 patients, the distance between the LV catheter position (defined as the geometric center of the segment it was found to be in), and the closest point of the epicardium that is estimated to activate at this time. The markers are 8 mm in diameter.

## Discussion

In this retrospective study based on 135 patients undergoing CRT, we estimated QLV using a standard 12-lead ECG. There was no statistical difference between non-invasively estimated QLV and QLV measured during the implant procedure. The median distance between measured and estimated LV pacing site was 18.6 mm (IQR 17.3 mm). However, the location of LV pacing site was known with segment-level precision; LV pacing site was always placed in the segment center. For reference, in the generic model, the mean epicardial area of the defined segments is 601 mm^2^. If the segments were perfectly square in shape, this would correspond to a side length of approximately 25 mm. This is an adequate approximation for all segments except the apex which is circular and even larger; it has a mean diameter of 47 mm.

We presented four phenotypes of bipolar electrograms and show from a theoretical standpoint how local activation should be measured differently in the four situations. To our knowledge, such categorization and presentation is novel.

**FIGURE 9:**
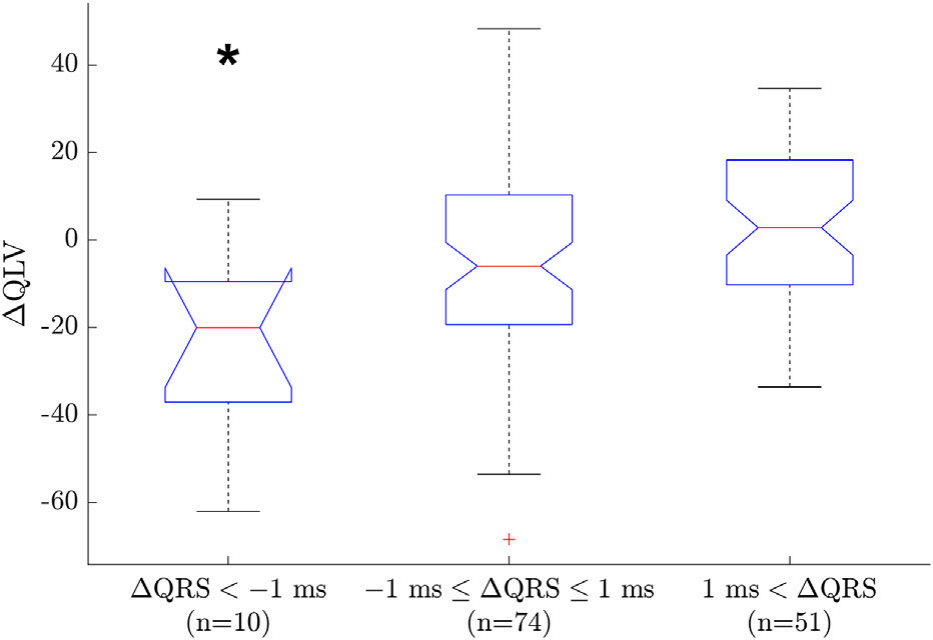
Boxplot between groups of $\Delta_{QRS}$ and $\Delta_{QLV}$ . The plot shows mean and IQR, with one outliers marked as a cross. The group representing QRS underestimation showed a statistically significant (negative) error in QLV estimation (*p < 0.05). This may be explained by the QRS duration imposing an upper bound on the QLV estimate.

Since we are using a generic geometry with uniform conduction velocity for the whole heart, it is not always possible to match the ECG exactly. One measure of the goodness of fit for the model is the error in matching QRS duration ( $\Delta_{QRS}$ ). We divided $\Delta_{QRS}$ into three groups representing underestimation of QRS ( $\Delta_{QRS}<-1\;ms$ ), exact estimation of QRS ( $-1\;ms\leq \Delta_{QRS}\leq +1\;ms$ ) and overestimation of QRS ( $\Delta_{QRS}>+1\;ms$ ). These groups were compared with a one-way ANOVA analysis, and the group representing QRS underestimation was significantly different from the other two ( p<0.05 ). Figure 9 shows a boxplot with median and IQR for each group. Since the QRS duration imposes an upper limit of the QLV estimate, it is not surprising that QRS underestimation also (on average) leads to QLV underestimation. However, it is only in four cases that QRS underestimation exceeds -4 ms, and these cases are the main drivers of this group. The largest $\Delta_{QRS}$ was 4 ms; for the 125 of 135 cases with $\Delta_{QRS}$ between -1 ms and +4 ms, there was no relation between $\Delta_{QRS}$ and $\Delta_{QLV}$ .

The varying response to CRT treatment between patients has led to an immense number of studies investigating both patient selection and optimal treatment. Positioning of the LV electrode has long been a main concern for response and is still reported to be associated with outcome (Kutyifa et al., 2018). Further it is suggested that having an overview of the myocardial substrate in terms of fibrosis and scarring (Butter et al., 2021), as well as myocardial activation and coronary venous anatomy before the procedure would be beneficial. This makes it possible to plan the procedure and to aim for placing the LV lead in a coronary sinus branch over a viable myocardial region with late electrical activation. There are several steps in this process. Firstly, the activation pattern of the heart needs to be mapped. This is the aim of this study. Secondly, information on myocardial scarring and cardiac venous anatomy obtained through cardiac imaging (e.g., magnetic resonance imaging and cardiac computed tomography) should be fused with the model. Then, the optimal pacing site can be determined based on simulations and be provided to the physician prior to CRT implantation. However, possible challenges with achieving a stable LV lead position with acceptable pacing thresholds and no phrenic nerve stimulation may still exist.

The largest drawback of the approach described above is that it works in research settings but is too elaborate for normal clinical practice. This is the reason we explore the use of generic models and use of the 12-lead ECG only. Our results indicate that QLV, and likely the entire activation sequence, may be estimated non-invasively from a standard 12-lead ECG, potentially solving the inherent problem that QLV cannot be measured until the patient is undergoing CRT implantation. However, The Mapper is not constrained in positioning the latest activating site. In practice, LV lead placement is limited by coronary vein anatomy, presence of scar tissue, or unintentional phrenic nerve pacing in which case the lead should be repositioned. This will inherently lead to differences between estimated and measured QLV. Still, knowing the activation sequence of the heart and hence the latest activating site may provide a more narrow and targeted area to map during LV lead implantation. Precision could possibly be improved by using more patient specific anatomical models obtained e.g. from statistical shape modeling, which could be done automatically or semi-automatically with little physician effort. This, however, requires a large library of segmented CT/MRI images. We are currently building such a library to enable this.

An intrinsic difficulty with CRT patients is that they often also suffer from ischemic heart disease (IHD) and have areas of myocardial scarring. Especially scarring is a poor tissue substrate for stimulation and should be avoided. In the present study we used a generic model and did not take ischemia or scarring into account. However, the precision was practically the same whether patients were considered to have ischemic heart disease or not (+IHD: $\Delta_{QLV}=+2\;ms$ , SD = 24 ms, median distance 22 mm, − IHD: $\Delta_{QLV}=-8\;ms$ , SD = 20 ms, median distance 21 mm). We expect the reason for this to be the implicit incorporation of ischemia or scarring through adjustment of the propagation velocity. Ischemia causes slower conduction than healthy tissue. When computing the activation sequence of the heart, the overall QRS duration must be matched, and thus ischemia is indirectly taken into consideration, however without location or extent. If data on scarring or ischemia is available, it would be possible to transfer this information to the generic model in a segment-wise manner. This will be investigated in a future study.

It is well known that the bipolar electrogram changes configuration depending on the position and angle to the wavefront, making measurement of local activation time difficult. The variations theoretically constitute a continuous spectrum of morphological changes. However, in practice we identified only four different phenotypes, corresponding to wavefronts along the axis or transverse to the axis of the bipolar lead, each in both directions. This is an important result that demonstrates the feasibility of measuring bipolar EGMs and how they can be used similarly to unipolar EGMs to measure local activation time consistently.

### Limitations

The Mapper is limited in that it has up to six initial activation sites that can change in timing and position, and a uniform (but anisotropic) conduction velocity. On this basis, the Mapper tries to match both the activation pattern (as governed by correlation between measured and simulated ECG) and QRS duration (by adjusting conduction velocity within certain physiological limits). Hence, it may be necessary to make a trade-off between the two. This is especially true in cases where the generic geometry is a poor match for the actual heart, and in cases where ischemia or scarring results in non-uniform conduction velocity. The limitation in the number of possible activation sites are most important in cases with complex activation sequences, where they may limit the accuracy of the solution.

## Conclusion

We demonstrated a novel non-invasive method for estimating QLV, based on the standard 12-lead ECG. On average, the method estimates myocardial activation and QLV with a small error, and may potentially be used to support patient selection, optimize implant procedures, and to simulate optimal stimulation parameters before the procedure. The use of a generic model has limitations that in some cases lead to considerable errors. This has to be taken into account when using The Mapper. Adding patient specific data, like electrode positions and body build might be necessary.

## Data Availability

Due to confidentiality agreements, supporting data cannot be made available.
